# Mechanical properties and DIC analyses of CAD/CAM materials

**DOI:** 10.4317/jced.53014

**Published:** 2016-12-01

**Authors:** Thiago Porto, Renato Roperto, Anna Akkus, Ozan Akkus, Sizenando Porto-Neto, Sorin Teich, Lisa Lang, Edson Campos

**Affiliations:** 1 DDS, MSc, PhD, Department of Restorative Dentistry, Faculty of Dentistry, Sao Paulo State University, Araraquara, SP–Brazil; 2 DDS, MSc, PhD, Department of Comprehensive Care, School of Dental Medicine, Case Western Reserve University, Cleveland, OH – USA; 3 PhD, Department of Comprehensive Care, School of Dental Medicine, Case Western Reserve University, Cleveland, OH–USA; 4 DDS, MBA, Department of Comprehensive Care, School of Dental Medicine, Case Western Reserve University, Cleveland, OH–USA

## Abstract

**Background:**

This study compared two well-known computer-aided-design/computer-aided-manufactured (CAD/CAM) blocks (Paradigm MZ100 [3M ESPE] and Vitablocs Mark II [Vita] in terms of fracture toughness (Kic), index of brittleness (BI) and stress/strain distributions.

**Material and Methods:**

Three-point bending test was used to calculate the fracture toughness, and the relationship between the Kic and the Vickers hardness was used to calculate the index of brittleness. Additionally, digital image correlation (DIC) was used to analyze the stress/strain distribution on both materials.

**Results:**

The values for fracture toughness obtained under three-point bending were 1.87Pa√m (±0.69) for Paradigm MZ100 and 1.18Pa√m (±0.17) for Vitablocs Mark II. For the index of brittleness, the values for Paradigm and Vitablocs were 73.13μm-1/2 (±30.72) and 550.22μm-1/2 (±82.46). One-way ANOVA was performed to find differences (α=0.05) and detected deviation between the stress/strain distributions on both materials.

**Conclusions:**

Both CAD/CAM materials tested presented similar fracture toughness, but, different strain/stress distributions. Both materials may perform similarly when used in CAD/CAM restorations.

** Key words:**Ceramic, CAD/CAM, hybrid materials, composite resin, fracture toughness.

## Introduction

The progress of dental technology has lead development of strong materials, such as ceramics, for indirect restorations. However, structural components of these materials have one obvious weakness: they tend to be brittle. Ceramics are brittle materials and susceptible to chipping. Although improvements in mechanical properties have been made during the past few years, some concerns still remain, such as fracture susceptibility to thermal and mechanical loading, and marginal misfit (1-3). Since the introduction of the first dental ceramic materials in the 19th century (4), different manufacturing techniques have been used in their fabrication. The traditional ceramic lost wax technique caused microcracks at the intaglio surface during the cooling phase. This is the reason why the technique was replaced for the porcelain-fused-to-metal (PFM) technique. Currently, metal-free ceramic restorations are being used because of advances in mechanical properties such as better material strength and better aesthetics (5,6).

Ceramics is by far the most common material used to fabricate CAD/CAM blocks (7), however, due to ceramic inherent characteristics as brittleness, low tensile strength, and low fracture toughness (8-10), in recent years new composite resin-based blocks have been developed (11,12). Resin composite CAD/CAM blocks seem to have on advantage over ceramic CAD/CAM blocks in terms of resilience and less abrasion on the antagonist enamel, helping to preserve overall balance of the dentition, and can also be easily adjusted or repaired (13). In contrast, all ceramic restorations have a high clinical failure rate in posterior sites (14,15), and are frequently replaced because of bulk fracture (2,16). Crack propagation of ceramic materials during mechanical deformation normally occurs differently from plastic materials (17).

However, CAD/CAM materials have not been fully investigated in terms of mechanical properties. The aim of this study was to compare the fracture toughness of CAD/CAM composite versus ceramic materials and to analyze the stress distribution through Digital Image Correlation (DIC) around the stress area. The null hypothesis is that there is no difference between fracture toughness, index of brittleness of both materials and no difference around the stress/strain concentration.

## Material and Methods

-Materials Selection

In this study, composite CAD/CAM block (Paradigm MZ100 – 3M ESPE, St. Paul, MN, USA) and feldspathic ceramic CAD/CAM block (Vitablocs Mark II –Vita Zahnfabrik, Essen, Germany) were used. The Paradigm MZ100 blocks are manufactured with the same 3M™ Z100™ composite resin restorative material. It has a highly polymeric matrix reinforced with 85 wt% of ultrafine (0.6μm) zirconia-silica ceramic particles that when synthesized, results in a resilient structure of nanocrystallinezirconia (18). In contrast, the CAD/CAM Vitablocs Mark II is manufactured with fine-particle (4μm) of feldsphatic ceramic uniformly embedded into a glassy matrix (19).  Table 1 shows the mechanical properties of both materials.

**Table 1:**
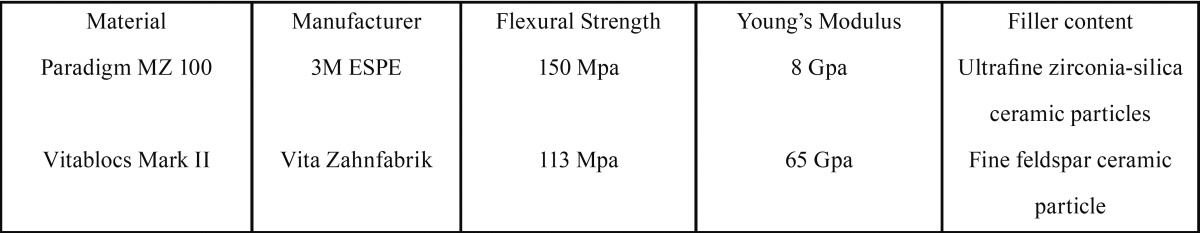
Mechanical properties and filler components.

Six composite and six ceramic CAD/CAM blocks (17.5mm x 14.8mm x 12mm) were first serially cut in rectangles (14.8mm x 12mm x 3mm) using a slow-speed saw (Isomet 1000; Buehler, Lake Bluff, USA), then cut again to create the beam size samples (14mm x 3.5mm x 3mm). In order to smooth out rough corners, beams were polished with a finishing and polishing machine (Ecomet 6, Buehler, Lake Bluff, IL, USA) using a sequence of sandpapers #240, #400, #800 and #1200. Final beam shape was confirmed with a digital calipers and had the dimensions of 14mm x 3mm x 2.5mm.

**Figure 1:**
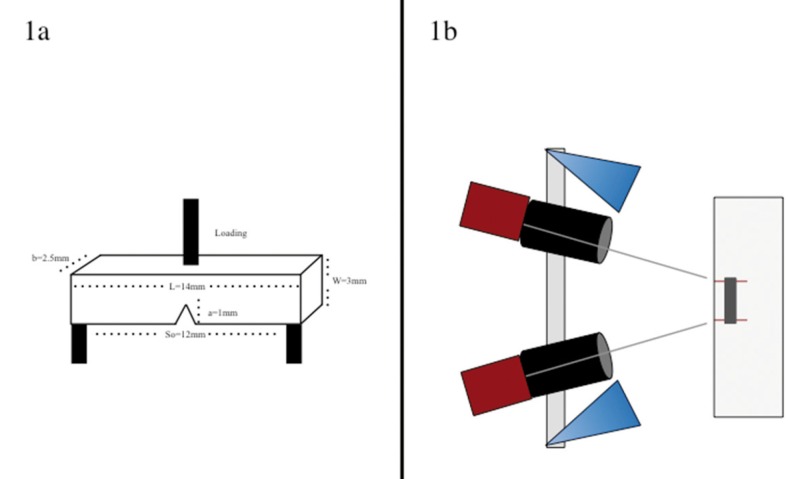
a) Schematic of beam samples positioned under three-point-bending test. b) Schematic of DIC camera lenses focused on beam samples.

The beam samples were polished, then analyzed by microscopy (Optical microscopy: 35x magnification, Nikon SMZ445, Melville, NY, USA) so every sample with any chipping defect was disposed of. After the selection procedure, beams were centrally positioned in circular plastic matrix molds of 16mm diameter and 10mm height, filled with microstoneplaster (Golden Type 3, Whip Mix, Louisville, KY, USA) and left for 15 min until it had set. In order to make the V-notch, specimens were positioned in the device by using special clips. The device had three axes: X-axis (horizontal) was where the ensemble was fixed with the clips; Y-axis (vertical) was connected a hand-piece (Henry Schein, Melville, NY, USA) with a diamond point 862-016FC (SS White, New Jersey, USA) needle shape. Finally, the V-notch was created using the tip of the diamond point along the Z-axis (width) with an approximate depth of 1.0mm (Fig. 1); further specimens were cleaned using deionized water in a sonic bath (Boekel Analog Model 139400, PA, USA) for 30min.

-Fracture toughness (three-point bending test) and index of brittleness

**Figure 2:**
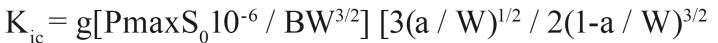
Equation 1.

**Figure 3:**
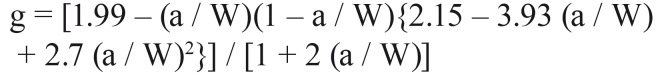
Equation 2.

The three-point bending test to calculate the fracture toughness was made following the methods given by ASTM C1421-10 ‘*Standard test methods for determination of fracture toughness of advanced ceramics at ambient temperature*’ (20). With the V-notch facing down, beam samples were positioned following the schematic in figure 1a. With a span (S0) of 12mm the three-pointing bend test was performed in a Universal testing machine (Test Resources, Shakopee, MN, USA) at a crosshead speed of 0.5mm/min until failure. The fracture toughness (Kic) of each material was calculated following equations (1) and (2): (Figs. 2,3).

In equation (1) Kic is the fracture toughness, while g is a function of the ratio *a/W*, *Pmax* is the maximum load (N) at the moment of the fracture, *B (m)* is the sample side to side parallel to the support, *W (m)* is sample top to bottom perpendicular to the support, and a *(m)* is depth of V-notch.

**Figure 4:**
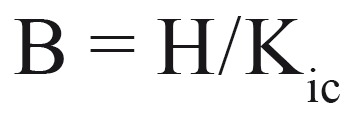
Equation 3.

The Brittleness index (BI) was calculated following the proposal by Lawn and Marshall, a derivation from Vickers hardness and (H) and fracture toughness (Kic) of the material (Eq. (3)), (Fig .4).

-DIC analyzes

**Figure 5:**
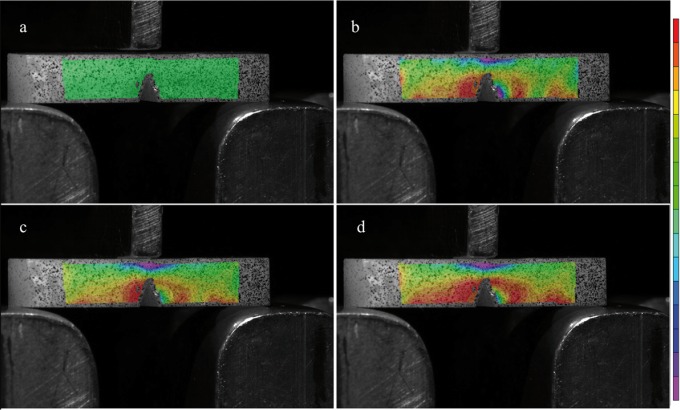
a) The first photographbefore the three-point bendingthe green area without strain/stress concentration is visible. b) On the second when the test starts some strain/stress concentration around the tip of the crack is visible. c) With more pressure on the specimen strain/stress concentration in front of the tip increases. d) The last photograph before failure show clearly the increase of the red area around the tip of the crack, moreover there is a big area of stress distribution at the bottom of the sample.

**Figure 6:**
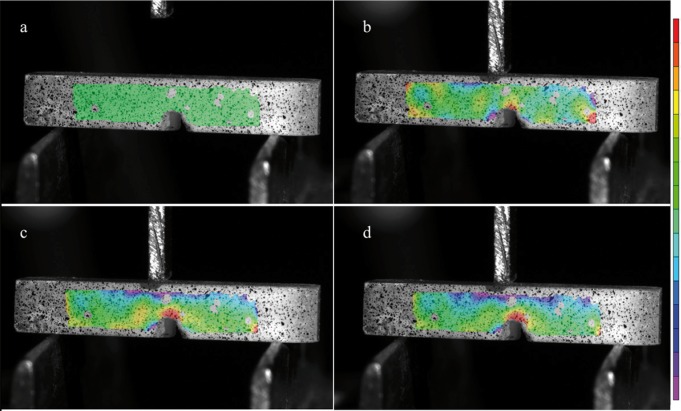
a) The first photograph before the three-point bending the green area without strain/stress concentration is visible. b) At the moment of the test starts, at the top of the crack tip a stress/strain concentration is seeable. c) As the pressure against the samples increase, the red area enhances showing the stress/strain concentration. d) The photo shows the moment before failure, and in contrast with composite resin there is not area of stress distribution at the bottom. The red area is concentrated at the top of the crack tip, probably due to different materials composition.

During the loading test, two calibrated 100mm F2.8 D macro lenses (Tokina, Tokyo, Japan) were positioned on a tripod, focused on the beam (Fig. 1b). Because direct frontal illumination can cause reflection from the sample surface, interfering with the reading, two sources of light were positioned at the right side of each macro lenses. Before the image capturing, lenses were calibrated and the sample surface was sputtered first with a white ink and 10min later with a black speckles layer as shown in figures 5 and 6.

One hundred and fifty photographs were taken during the loading of the each specimen and analyzed on two computer softwares (VIC – Snap 8 and VIC – 3D, Correlated Solutions, USA). Only four images were chosen from each sample, one at the beginning of the three point bending test, two in the middle, and one just before failure. The pictures showed different color concentrations from red (most stress concentration) to purple (compressive concentration).

-Statistical analyses

Statistical analyzes were carried out using SPSS version 23.0 (SPSS, IBM Corp., Chicago, IL, USA) and *p*<0.05 was considered significant. Both materials were first applied to Shapiro-Wilk’s test and then to Levene’s test, respectively, for normal distribution and homogeneity of variances. Further, the values obtained for each group were analyzed by one-way ANOVA.

## Results

-Fracture toughness (Kic) and index of brittleness test:

Values for fracture toughness (Kic) and Brittleness Index (BI) are reported in  Table 2. For both materials analyzed in this study, statistical findings showed normal distribution for data through Shapiro-Wilk’s test, and also Levene’s test showed homogeneity of variances.

**Table 2:**
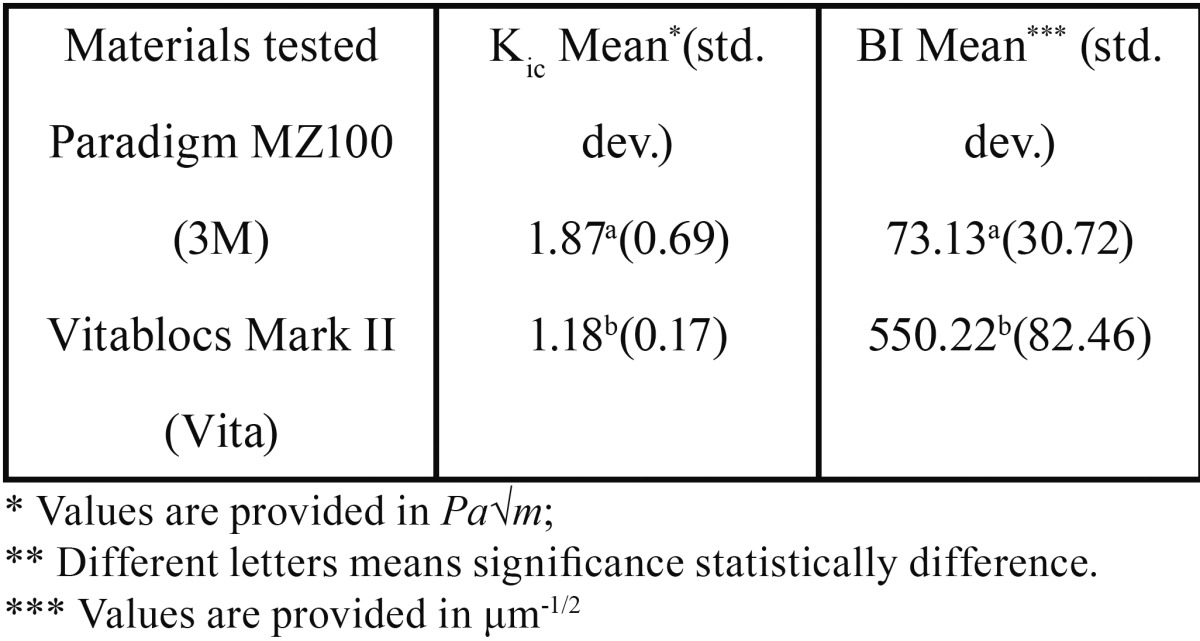
Descriptive statistics from statistical findings.

With the lower Kic value between two materials, the Vitablocs Mark II had the higher BI, which means that composite resin CAD/CAM block has a better machinability than the ceramic block. One-way ANOVA showed that there was a significant statistical difference between both variables among the materials (*p*<.05).

-Digital Image Correlation (DIC) test

The DIC test for Paradigm MZ100 is shown in figure 5 and for Vitablocks Mark II is shown in figure 6. The strain/stress concentration is shown in front of the crack tip, whereas the red color means the highest strain concentration, while the purple is the compressive strength.

## Discussion

This study aimed to test two well-know CAD/CAM materials, comparing the fracture toughness, index of brittleness, and stress distribution through Digital Image Correlation (DIC). The first null hypothesis was that no difference between fracture toughness and index of brittleness of both materials would occur. The second null hypothesis was that no difference around the stress/strain concentration would be found. Both null hypotheses have been rejected.

The fracture toughness (Kic) of Paradigm MZ100 shows a higher value compared with Vitablocs Mark II; they were considered statistically different after performing the analysis of variance. This was probably due to different compositions. Made from 3M™ Z100™ restorative material, the composite resin CAD/CAM block has ultrafine zirconia-silica ceramics particles combined with the polymeric matrix bisGMA and TEGDMA as disclosed by the manufacturer. In contrast, the Vitablocs Mark II is composed of fine feldspar ceramic particles with no inorganic phase according to manufacturer specifications, causing it to have a less dissipative characteristic. Our findings show that Paradigm MZ100 has a better resistanceto pre-crack when compared to feldspathic glass ceramic. Similar findings have been reported by other authors when glass ceramics was compared with hybrid composite resin blocks (11). Furthermore, the wearing process that occurs during mastication is also important, and material fatigue will start as soon as the first cracks begin to show up on the occlusal surface (2). On composite resin materials a softening effect is induced by hydrolysis and will accelerate the *in vivo* wear of the composite resin block (21). Therefore, to create a stronger material, the manufacturers have increased the amount of inorganic fillers, which are incorporated in the resin matrix (12,15) (e.g. Paradigm MZ100 has 85wt% ultrafine zirconia-silica ceramic particles).

The index of brittleness of Vitablocs Mark II shows a higher value when compared to Paradigm MZ100, and they were also considered statistically different after performing the analysis of variance. The brittleness of ceramics materials differs from composite resin blocks behavior due to its filler contents; our results for index of brittleness calculation were comparable to similar findings in the literature (9,17). Furthermore, the index of brittleness depends on properties and material microstructure and has been labeled by different authors as the materials’ machinability performance. The Vickers hardness and fracture toughness are derived to calculate the index of brittleness and they could be considered as volume-controlled and surface-controlled, respectively. The index of brittleness shows that Paradigm MZ100 is less susceptible to chipping and surface flaws during milling, which is one of the consequences of the computer-aided machining process (9).

The arrays of CAD/CAM ceramics are subject to chipping and surface flaws, regardless of the material’s composition. Clinicians should keep in mind these negative effects that could lead the restorations to premature failure, particularly when conservative tooth reduction and beveled margins are a necessity in a cavity preparation (3,9).

Moreover the materials also show different stress/strain concentration along the beam samples (Figs. 5,6). The digital image correlation analysis emphasize what happens during fracture toughness determinations. These materials perform differently under compression; the dissipative characteristic of composite resin is much higher than that of glass ceramic. The Kictest (materials’ ability to resist a crack and further failure) seems to be more suitable than flexural strength tests (7,12,22-24). An experiment conducted with a pre-cracked beam can actually present many advantages on this approach, such as: (i) suitability for brittle materials, ceramics, hybrid ceramic and composite resin; (ii) constant crack growth can be monitored in real-time; (iii) potential to study the ceramic resistance reaction as well as the macrocrack route by the DIC analysis; (iv) the v-notched samples are easier to prepare when compared to flexural strength tests; (v) the maximum load is lower when compared to flexural strength three-point bending test (25).

One of the limitations of this study was the difficulty in getting photographs during the crack propagation. For both materials, the failure occurred suddenly and no pictures during the crack propagation were taken.

## Conclusions

At present, restoration with CAD/CAM materials are not always successful because of material’s limitation and indication; it is a recurrent problem that every Prosthodontist is faced with. In this study, we found that something relatively minor, such as the fracture toughness and stress distribution of two common CAD/CAM materials that can make a real difference into the real clinical application of these materials. Our research could serve as a baseline to test different classes of materials, such as hybrid ceramics and zirconia, in order to confirm the longevity of these.
